# One-Step Synthesis of Metal/Oxide Nanocomposites by Gas Phase Condensation

**DOI:** 10.3390/nano9020219

**Published:** 2019-02-06

**Authors:** Nicola Patelli, Andrea Migliori, Vittorio Morandi, Luca Pasquini

**Affiliations:** 1 Department of Physics and Astronomy, Alma Mater Studiorum Università di Bologna, Viale Berti-Pichat 6/2, 40127 Bologna, Italy; 2 Section of Bologna, Institute of Microelectronics and Microsystems, National Research Council, Via Gobetti 101, 40129 Bologna, Italy; migliori@bo.imm.cnr.it (A.M.); morandi@bo.imm.cnr.it (V.M.)

**Keywords:** nanoparticles, nanocomposites, gas phase condensation, electron microscopy, metal oxides, alloys, iron, cobalt, titanium

## Abstract

Metallic nanoparticles (NPs), either supported on a porous oxide framework or finely dispersed within an oxide matrix, find applications in catalysis, plasmonics, nanomagnetism and energy conversion, among others. The development of synthetic routes that enable to control the morphology, chemical composition, crystal structure and mutual interaction of metallic and oxide phases is necessary in order to tailor the properties of this class of nanomaterials. With this work, we aim at developing a novel method for the synthesis of metal/oxide nanocomposites based on the assembly of NPs formed by gas phase condensation of metal vapors in a He/O_2_ atmosphere. This new approach relies on the independent evaporation of two metallic precursors with strongly different oxidation enthalpies. Our goal is to show that the precursor with less negative enthalpy gives birth to metallic NPs, while the other to oxide NPs. The selected case study for this work is the synthesis of a Fe-Co/TiO_x_ nanocomposite, a system of great interest for its catalytic and magnetic properties. By exploiting the new concept, we achieve the desired target, i.e., a nanoscale dispersion of metallic alloy NPs within titanium oxide NPs, the structure of which can be tailored into TiO_1-δ_ or TiO_2_ by controlling the synthesis and processing atmosphere. The proposed synthesis technique is versatile and scalable for the production of many NPs-assembled metal/oxide nanocomposites.

## 1. Introduction

Oxide-supported metal nanoparticles (NPs) are a class of functional materials that find innovative applications in many materials science fields such as catalysis for the production of synthetic hydrocarbons [1,2,3] and CO reduction [4], chemical synthesis [5,6], nanoplasmonics [7] for the development of higher efficiency photovoltaic cells [5,8], and magnetism [9]. The presence of the oxide support within the nanocomposite does not only affect the size and shape of metal NPs [10,11], but is also crucial to prevent coarsening and sintering [12], and is often responsible for a radical change in physical properties because of electronic interactions at interfacial sites [4,13,14].

In the last decade, much effort has been spent to develop novel and flexible synthesis routes for metal/oxide nanocomposites (NCs). Most of these techniques involve two-step processes, in which a porous oxide host (typically zeolites, Al_2_O_3_) or oxide NPs [15] are imbued with a colloidal suspension of metallic NPs produced via physical (e.g., pulsed laser ablation in liquid) or chemical methods (e.g., precipitation and nitride impregnation). Metal NP encapsulation into oxide shells [16] and in pores and channels of hierarchical zeolites has been also reported but, albeit innovative, these approaches do not convey a homogeneous distribution of the supported NPs and are limited to a small range of materials [17]. Mechanochemistry via ball milling followed by suitable thermal treatments can be successfully applied to the synthesis of metallic NPs in an oxide matrix [18]. However, it is not possible to control the morphology of the metal and oxide particles independently, and ductile materials are very difficult to process. Metallic NPs embedded in an oxide matrix can be prepared by a sol–gel method [19,20], again with some limitations on the independent control of the two phases. The deposition of metallic NPs on oxide surfaces is of great importance for fundamental studies on model systems [21], but cannot be used for the synthesis of 3D bulk materials.

In this work, we present a novel one-step strategy for the synthesis of metal/oxide NCs through the physical assembly of NPs. More specifically, NPs are formed by gas phase condensation of metallic vapors in a He/O_2_ mixed atmosphere. Two metallic precursors with different oxidation enthalpies are evaporated simultaneously and independently; the one with less negative enthalpy forms metallic NPs, while the other provides the seed for oxide NPs. Thermal treatments in suitable atmosphere can be further applied to modify structure and morphology. We apply this concept to the synthesis of a Fe-Co/TiO_x_ NC. We also demonstrate how the stoichiometry and crystalline structure of TiO_x_ can be tailored by controlling the O_2_ partial pressure during the synthesis and processing atmosphere. The presented method is general and scalable for production of 3D oxide-supported metal NPs.

## 2. Materials and Methods

**Figure 1:**
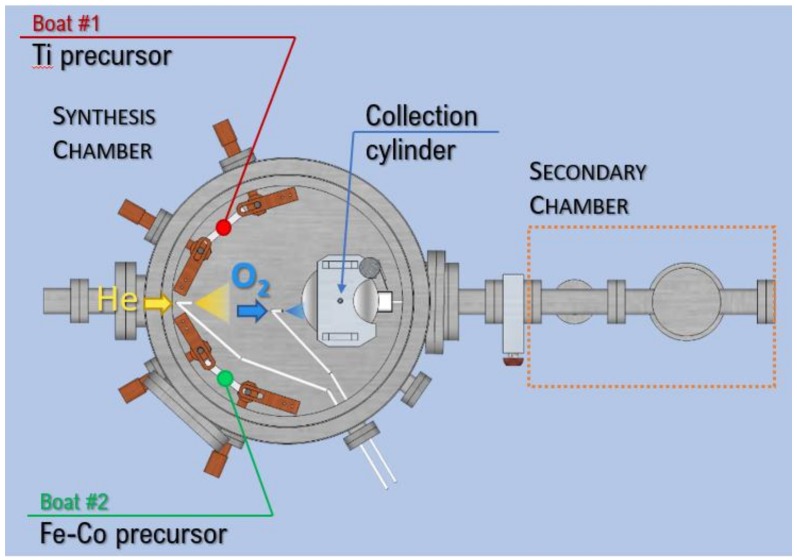
Top view of the GPC apparatus.

Fe-Co/TiO_x_ nanocomposites (NCs) were grown by gas phase condensation (GPC) in an ultra-high vacuum (UHV) chamber starting from Ti (99.9%), Fe (99.9%) and Co (99.9%) powders. A schematic sketch of the system is shown in Figure 1. The main chamber is equipped with two thermal evaporation sources (Joule-heated tungsten boats). Individual evaporation rates can be monitored using a quartz crystal balance positioned close to the collection cylinder.

During the synthesis, He (99.9996% purity) and O_2_ (99.9999% purity) are fed into the chamber (previously evacuated to 2 × 10^−5^ Pa) using two mass flow controllers, while the total pressure is maintained at 260 Pa by means of a rotary pump. In all the experiments reported here the O_2_ content in the atmosphere was kept below 1 mol.%. The precursor materials are evaporated slightly above their melting point. NPs nucleation takes place in the gas phase where metal vapors rapidly supersaturate because of thermalization with the He/O_2_ atmosphere. The NPs are collected onto the rotating stainless-steel cylinder filled with liquid N_2_. Finally, the NPs are scraped off the cylinder and transferred into the secondary UHV chamber, which is equipped with an independent pumping system. Here it is possible to press the NPs into a pellet and/or to perform thermal treatments under vacuum or controlled atmosphere.

The average elemental composition of the NCs was determined using a Leica Cambridge Stereoscan 360 scanning electron microscope (SEM) equipped with Oxford Instruments X-ray detector for energy dispersive X-ray microanalysis (EDX) (Oxford Instruments, Abingdon-on-Thames, UK). X-ray diffraction (XRD) patterns were collected using a PANalytical X’celerator powder diffractometer (Malvern Panalytical, Malvern, UK) employing Cu Kα radiation (λ = 1.5406 Å). The patterns were recorded under ambient air in about 30 min. Quantitative analysis based on the Rietveld method was carried out with the MAUD program [22] to determine the lattice parameters, crystallite size and phase abundance.

Elemental mapping and phase distribution at the nanoscale were investigated with a FEI Tecnai F20 ST transmission electron microscope (TEM) (FEI Company, Hillsboro, OR, USA). EDX profiling and elemental mapping with a spatial resolution of 2 nm were recorded in scanning transmission mode (STEM) at 200 kV. The crystalline phase distribution was determined by operating in selected area diffraction (SAD) and High Resolution (HR-TEM) mode. For TEM analysis, the samples were dispersed in isopropanol, sonicated and the NPs suspension was drop-casted on a holey carbon grid.

### 2.1. Synthesis of NPs and NCs Samples

#### 2.1.1. TiO_x_ NPs

This work can be divided in three main parts, the first regarding the influence of O_2_ content in the atmosphere and of post-synthesis treatments on the stoichiometry and structure of Ti oxide NPs (from here indicated TiO_x_ NPs). Two samples, named Ti-O_l and Ti-O_h, were synthetized at O_2_ partial pressures of 0.4 and 2.2 Pa, respectively. Table 1 lists the conditions applied during the synthesis and the successive treatments. The thermal treatments were performed either in H_2_ (99.995% purity), Ar (99.999% purity) or air in a tubular stainless-steel oven at T = 400 °C for 24 h.

#### 2.1.2. Fe-Co Alloy NPs

The second part of this work concerns the synthesis of Fe-Co alloy NPs in a He atmosphere. To this purpose, one thermal source is loaded with the desired mixture of Fe and Co powders. The powders are melted under high vacuum and rapidly cooled to about 1000 °C in order to homogenize the alloy precursor. Four samples with composition Fe_100-x_Co_x_ (with x = 0, 23, 48, 68 at.%) were prepared. Table 2 lists the Fe and Co content of all as-prepared NPs as determined from EDX analysis.

We notice that the NPs composition is compatible (within the uncertainties) with the precursor composition for all samples but the Co-richest one. For the Fe-Co system, the stoichiometry can be quite well preserved because Fe and Co have similar vapor pressures. Nevertheless, the slightly higher evaporation rate of Fe [23] can be the reason for the small increase in the Fe content noticeable in Table 2 for the Co-richest sample.

#### 2.1.3. Fe/TiO_x_ and Fe-Co/TiO_x_ NCs

To obtain Fe_100-x_Co_x_ NPs (x = 0, 50) supported on TiO_x_ NPs, the evaporation chamber was equipped with two tungsten boats (as shown in Figure 1). The boats were separated by ~30 cm in order to avoid the mixing of metal vapors before NPs nucleation [24], which may lead to the nucleation of a ternary Ti-Fe-Co alloy. The synthesis parameters are reported in Table 3. The NCs were grown in a He atmosphere with the same O_2_ content as for the Ti-O_l sample. The individual evaporation rates of Fe-Co and Ti were monitored with the quartz crystal balance and tuned in order to obtain a Fe-Co content in the NCs of about 10 wt%. The relative Fe/Co content in the NCs determined by SEM-EDX was consistent with the precursor within the uncertainties.

## 3. Results and Discussion

### 3.1. TiO_x_ NPs

**Figure 2:**
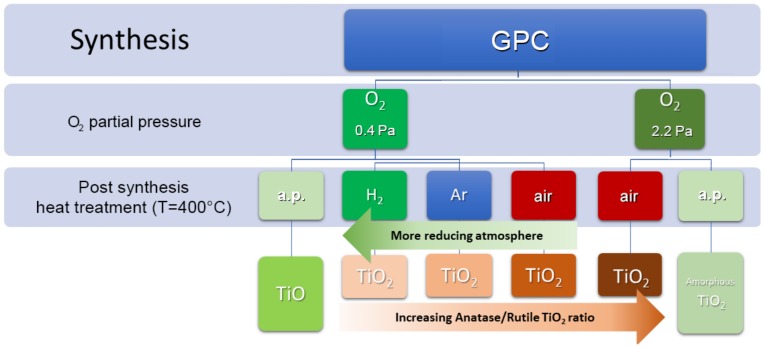
Schematics of the phases identified in TiO_x_ NPs as a function of the conditions applied during synthesis by GPC and successive thermal treatments.

The synthesis conditions, as well as the post-synthesis treatments, turn out to have a great influence on the structure and phase of the TiO_x_ NPs, as summarized in Figure 2.

#### 3.1.1. As-Prepared Samples

**Figure 3:**
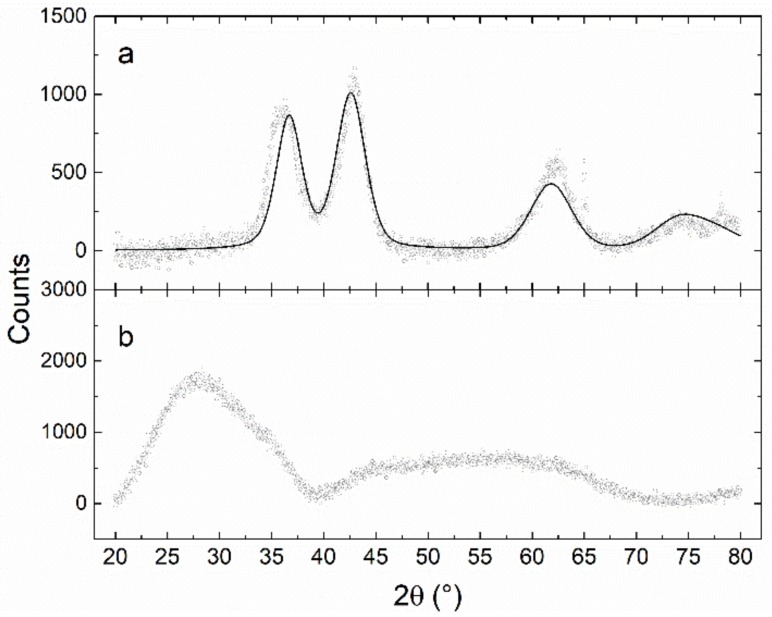
XRD patterns of as-prepared TiO_x_ NPs. (**a**) Ti-O_l; (**b**) Ti-O_h. The grey circles represent experimental data. The result of Rietveld refinement is shown for Ti-O_l as a black solid line.

Figure 3 shows that the structure of the as-prepared TiO_x_ NPs strongly depends on the O_2_ content in the atmosphere. At low O_2_ (sample Ti-O_l) crystalline Ti monoxide (TiO_1-δ_) NPs are obtained (Figure 3a). TiO_1-δ_ has a disordered nonstoichiometric rocksalt structure that can exist over a wide compositional range, from δ=0.30 to δ=−0.25, due to the presence of vacancies in both the Ti and O sub-lattices [25,26,27]. It is kinetically stable up to about 400 °C.

The broadening of TiO_1-δ_ XRD peaks in Figure 3a is due to the small crystallite size $d_{\mathrm{TiO}}$ and to the high root-mean-square microstrain $\varepsilon_{rms}$. The relative height of the peaks is strongly influenced by the occupancy factor of Ti and O sub-lattices and permits to estimate the average stoichiometry of the NPs. From the quantitative Rietveld analysis, we obtained $d_{\mathrm{TiO}}=9\pm \;$2 nm, $\varepsilon_{rms}\approx 2\;\%$, and δ= 0.25± 0.05, i.e., an oxygen-deficient stoichiometry. However, the high microstrain points to a nonhomogeneous stoichiometry across the sample. In fact, the poor quality of the fit in Figure 3a indicates that a model with a single value of δ and $d_{\mathrm{TiO}}$ does not represent satisfactorily the structure of TiO_1-δ_ NPs. We suggest that the δ value may experience significant fluctuations among the NPs depending on their diameter and on small variations in the vapor pressure during the synthesis.

At higher O_2_ content in the synthesis atmosphere (sample Ti-O_h), two broad humps indicate the formation of amorphous TiO_2_ (Figure 3b) [28], while the Bragg reflections of TiO_1-δ_ are not detected.

#### 3.1.2. Post-Synthesis Thermal Treatment

**Figure 4:**
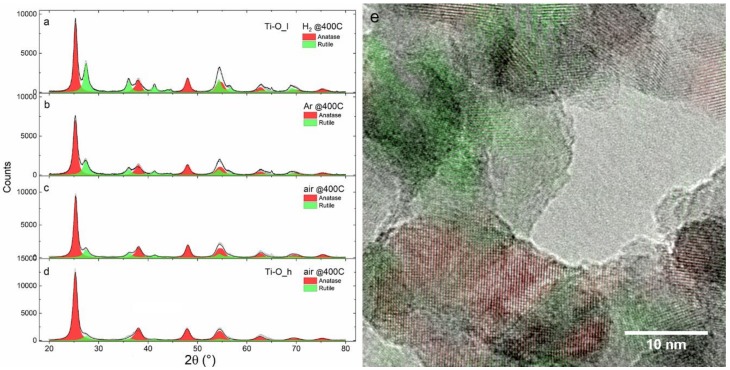
XRD patterns of TiO_x_ NPs samples after a thermal treatment at 400 °C. (**a**–**c**) sample Ti-O_l in (**a**) H_2_, (**b**) Ar, and (**c**) air; (**d**) sample Ti-O_h in air. The results of Rietveld refinement are reported in Table 4; (**e**) HR-TEM image of sample Ti-O_l treated in air (corresponding to pattern [c]) with the anatase and rutile fringes highlighted in red and green, respectively.

Figure 4 displays the XRD patterns of TiO_x_ NPs subjected to thermal treatments at T = 400 °C. In sample Ti-O_l, the Bragg reflections of TiO_1-δ_ disappear in favor of those associated with rutile and anatase TiO_2_ (Figure 4a–c). The anatase content (see Table 4) increases with the oxidative power of the atmosphere ranging from 56 ± 1 wt% (in H_2_) to 74 ± 2 wt% (in air). The previously amorphous sample Ti-O_h appears completely crystallized and exhibits the highest anatase content (84 ± 1 wt%). The crystallite size of anatase is not significantly influenced by the treatment conditions and varies in the 13–16 nm range. The crystallite size of rutile is smaller (6–11 nm) and appears negatively correlated with the rutile content. The HR-TEM image in Figure 4e reveals the coexistence of rutile and anatase polymorphs on a nanoscale level and confirms that no residual metallic Ti is left after the treatments, in agreement with the results of previous X-ray absorption experiments [28].

The results related to synthesis and processing of TiO_x_ NPs can be rationalized as follows. If the O_2_ content in the synthesis atmosphere is too low (sample Ti-O_l), full oxidation of the nucleated Ti NPs into TiO_2_ is not possible, and non-stoichiometric TiO_1-δ_ is obtained. This is not because the initial O_2_ partial pressure (0.4 Pa) is below the equilibrium pressure for TiO_2_ formation (which is ridiculously low, i.e., ~10^−59^ Pa at 400 °C and ~10^−150^ Pa at room temperature) but because there is not enough O_2_ available. The freshly evaporated Ti consumes almost all the O_2_ and the competition between the evaporation rate and the O_2_ inlet flow rate dictates the final stoichiometry. Therefore, it should be possible to tailor the off-stoichiometry δ by playing with these parameters; this may be the subject of future experiments. It is also worth noticing that the TiO_1-δ_ NPs obtained in this way are kinetically stable at room temperature, i.e., they are not oxidized into TiO_2_ upon exposure to ambient air.

Conversely, at sufficiently high O_2_ inlet flow (sample Ti-O_h), it is possible to achieve (almost) full oxidation of the NPs. It is well known that amorphous TiO_2_ is obtained when oxidation is carried out close to room temperature, whereas oxidation above 350 °C leads to crystalline TiO_2_ [29]. This suggests that NPs oxidation takes place after cool down from the evaporation temperature has taken place via thermalization with the surrounding He gas. In agreement with the vast literature on TiO_2_, crystallization of amorphous NPs is induced by a thermal treatment in air at 400 °C [28,30].

The formation of crystalline TiO_2_ by heating the TiO_1-δ_ NPs in Ar or H_2_ is less straightforward, but can be understood from thermodynamic data considering the presence of water vapor impurities. Let us take into account the following reaction, pertinent to the treatment in H_2_:

(1) $$\mathrm{TiO}\;+\;H_{2}O\;↔\;{\mathrm{TiO}}_{2}\;+\;H_{2}$$

The ratio between the water vapor pressure P_H2O_ and hydrogen pressure P_H2_, at which reaction (1) is at equilibrium, can be easily calculated using the van ‘t Hoff equation, yielding:

(2) $${\left(\frac{P_{H2O}}{P_{H2}}\right)}_{eq}=\exp\left(\frac{\Delta H_{TiO2}-\Delta H_{H2O}}{2RT}\right)$$

where $\Delta H_{TiO2}=-853$ kJ/mol O_2_ is the enthalpy of TiO oxidation, i.e., of the reaction 2TiO + O_2_→2TiO_2_, and $\Delta H_{H2O}=-495$ kJ/mol O_2_ is the enthalpy of water formation, 2H_2_ + O_2_→2H_2_O. With these data and T = 673 K, Equation (2) yields ${\left(P_{H2O}/P_{H2}\right)}_{eq}\approx 10^{-14}$. Since the water content due to impurities in the gas and to desorption from the reactor walls is certainly higher than that, we must expect that reaction (1) proceeds rightwards, i.e., that oxidation of TiO_1-δ_ NPs takes place during the thermal treatment. Notice that this behavior arises from the strongly negative formation enthalpy of TiO_2_; oxides of late transition metals such as Fe and Co, on the contrary, would be reduced under the same atmosphere. This is a key factor in the processing of NCs samples, as we will show later on.

The oxidative power of the treatment atmosphere influences the rutile to anatase ratio. This is also in agreement with the literature, which shows that oxygen-deficient conditions favor the formation of rutile [30], because rutile itself exhibits a slightly oxygen-deficient stoichiometry.

### 3.2. Fe and Fe-Co Alloy NPs

**Figure 5:**
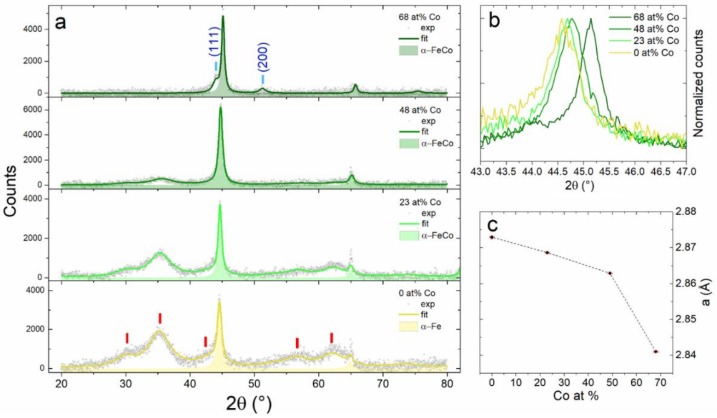
(**a**) XRD patterns of Fe_100-x_Co_x_ samples (x = 68-0 from top to bottom). Grey circles represent experimental data, while the colored solid lines are the best fits. The colored area highlights BCC peaks of the alloy, while colored vertical bars marks the Bragg reflections of cobalt ferrite (red) and FCC Fe-Co alloy (cyan); (**b**) A detail of the experimental data showing the shift of the (110) BCC reflection with increasing Co content; (**c**) Lattice parameter *a* obtained from Rietveld refinement (Table 5) as a function of Co content, with the error bars are displayed in red.

Figure 5a shows the XRD patterns of the as-prepared Fe_100-x_Co_x_ NPs. The mean crystallite size *d* and lattice parameter of all identified phases are reported in Table 5. In all samples, we observe the (110) and (200) Bragg reflections characteristic of a body-centered cubic (BCC) α-phase. Only in the Co-richest sample Fe_32_Co_68_, the (111) and (200) Bragg reflections of a face-centered cubic (FCC) γ-phase are clearly visible. According to the Fe-Co phase diagram [31], Fe and Co are completely miscible at room temperature up to 72 at.% Co content forming a BCC α-phase, while FCC and BCC phases coexist at higher Co content (72 to 92 at.% Co). Here we observe the FCC γ-phase already at 68 at.% Co. This deviation with respect to the bulk phase diagram [31] in nanoalloy systems is due to the increase in relative magnitude of surface energy and is size-dependent [10]. FCC and BCC coexistence in Fe-Co nanoalloys has already been reported above 42 at.% Co for NPs of 15 nm [32].

The (110) reflection of the α-phase (Figure 5b) shifts towards higher angles with increasing Co content. This corresponds to the shrinking of the lattice parameter *a*, as plotted in Figure 5c, and is consistent with the smaller atomic radius of Co with respect to Fe.

**Figure 6:**
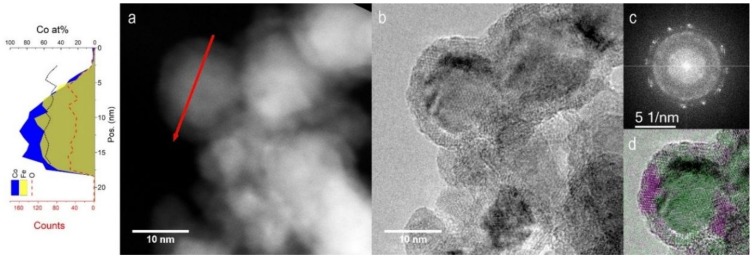
TEM analysis of Fe_52_Co_48_ NPs. (**a**) HAADF-STEM image and the corresponding EDX profile taken along the red arrow. The blue and yellow areas and the red dashed line represent Co, Fe and O X-ray fluorescence counts. The black solid line is the Co/(Co+Fe) atomic ratio within the NP; (**b**) HR-TEM image of the same area together with its (**c**) Fast-Fourier-Transform (FFT); (**d**) Mapping of crystal phases obtained from the analysis of planar spacing. α-FeCo (110) and cobalt ferrite (311) planes are represented in green and purple, respectively.

The broad peaks in the XRD patterns correspond to a magnetite (Fe_3_O_4_)-like spinel structure with an ultrafine crystallite size of 2–3 nm. This oxide forms when the NPs get in contact with the ambient air for XRD measurements. If air exposure is fast, the NPs begin to glow and oxidize completely. Conversely, if the exposure is sufficiently slow, as in this case where air flows into the sample holder gradually in about one hour, full oxidation can be avoided through the formation of a protective oxide shell [33,34]. The metal core / oxide shell morphology of the NPs gradually exposed to the air is confirmed by TEM investigations. The High Angle Annular Dark Field (HAADF) STEM image in Figure 6a shows a detail of Fe_52_Co_48_ sample. The NPs are surrounded by a lower contrast shell about 3 nm thick. This is a first indication in favor of the oxide nature of the shell. In fact, the contrast in incoherent HAADF-STEM images is proportional to *tZ*^1.7^, where *t* is the thickness and *Z* is the average atomic number. Further information on the composition of the shell is provided by the STEM-EDX profile of a single NP recorded along the red line in Figure 6a. The shell corresponds to the regions, in which the Co and Fe fluorescence counts (blue and yellow areas) start to decrease while the O counts (red dashed line) are still constant. Interestingly, the Co/(Co + Fe) atomic ratio (black solid line) is similar in the core and the shell. This result demonstrates that the oxide is actually a cobalt ferrite (Fe_100-x_Co_x_)_3_O_4_ with a magnetite-like spinel structure [35] and a Co/Fe ratio similar to that of the core.

The HR-TEM image of the same NPs in Figure 6b highlights the atomic-level structure of core and shell regions. The Fast Fourier Transform (FFT) operated over the HR-TEM image is displayed in Figure 6c. The bright spots correspond to α-Fe-Co (110) planes, while the inner broad ring is due to the (311) crystalline planes of cobalt ferrite. These contributions can be separated by applying a filter to the FFT image followed by an inverse transformation. In this way, one obtains two separate images for the lattice planes of the two phases. In Figure 6d, the separate images are superposed in false colors to the HR-TEM image. The α-Fe-Co lattice planes (green) are clearly visible in the core, while the cobalt ferrite planes (violet) belong to the shell.

Finally, the intensity of the oxide broad peaks in Figure 5a shows that the oxidation resistance upon air exposure augments with increasing Co content. A remarkable stability against oxidation was also pointed out for Fe_100-x_Co_x_ NPs with x≈40−50 prepared by hydrothermal synthesis [36].

### 3.3. Fe/TiO_x_ and Fe-Co/TiO_x_ NCs

**Figure 7:**
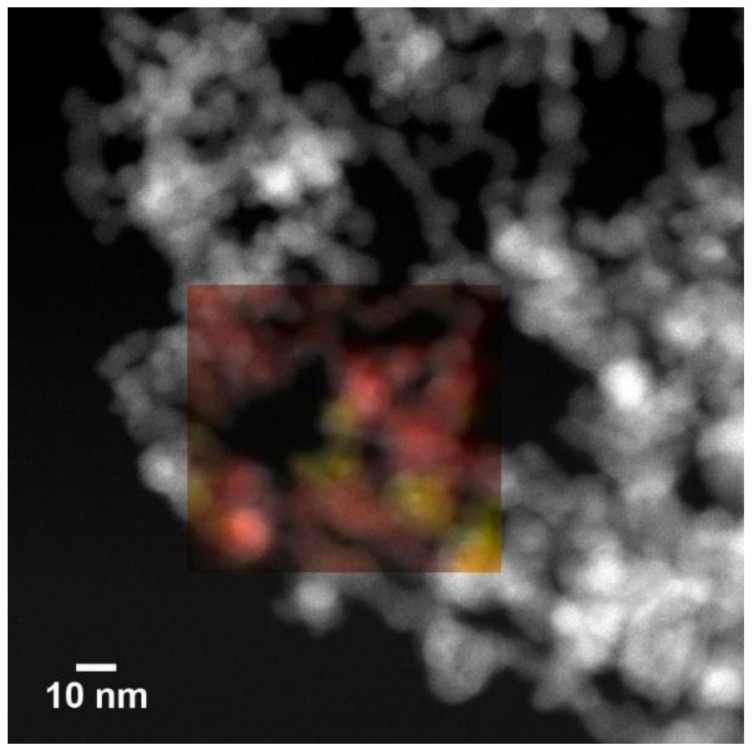
STEM-EDX elemental map of Fe (yellow) and Ti (red) in the as-prepared Fe/TiO_x_ NC.

The STEM-EDX map in Figure 7 (superimposed to the corresponding STEM image) highlights the nanocomposite nature of the NPs assembly obtained by co-evaporation of Fe and Ti. The elemental distribution of Fe (yellow) and Ti (red) clearly shows the good intermixing at the nanoscale level of Fe-rich and Ti-rich NPs.

**Figure 8:**
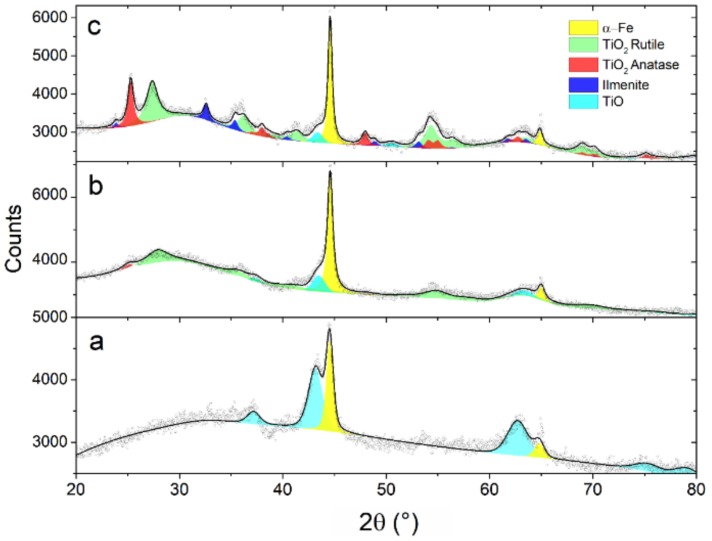
XRD patterns of Fe/TiO_x_ NCs. (**a**) As-prepared sample; (**b**,**c**) After thermal treatment in a H_2_ atmosphere, 1 MPa, for (**b**) 4 h and (**c**) 24 h. The colored areas highlight the contributions of different phases to the Rietveld refinement. Grey circles represent experimental data, while the black solid line shows the overall best fit.

**Figure 9:**
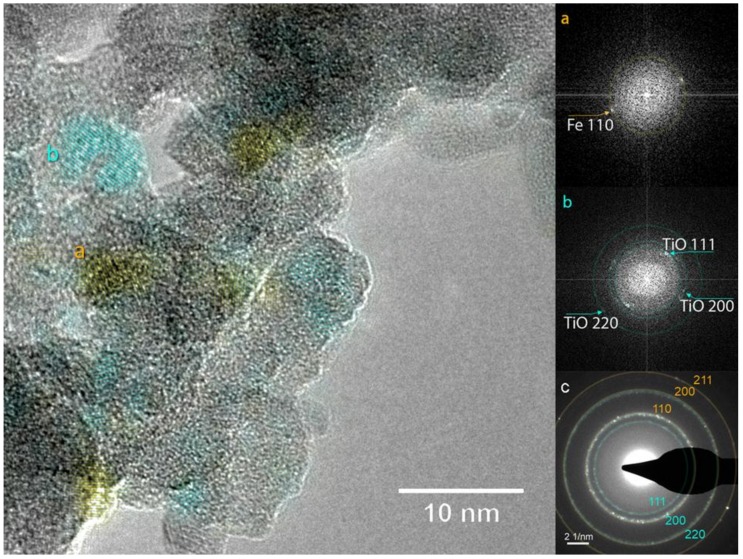
HR-TEM image of the as prepared Fe/TiO_x_ NC (left) and FFTs performed on selected areas labeled with lowercase letters showing the presence of (**a**) Fe and (**b**) TiO_1-δ_ lattice planes; (**c**) SAD pattern: Fe and TiO_1-δ_ lattice planes are labeled in yellow and cyan, respectively (yellow and cyan rings serve as guides for the eye). In the HR-TEM image, the lattice planes of Fe and TiO_1-δ_ are highlighted using the same colors.

According to the XRD pattern (Figure 8a), the as-prepared sample is constituted by α-Fe and TiO_1-δ_. This view is confirmed by TEM analysis, which also provides further clues on the nanoscale phase distribution. The HR-TEM image in Figure 9 and the FFTs performed over the regions labeled a and b show the intimate contact between NPs constituted by α-Fe (region a) and TiO_1-δ_ (region b).

**Figure 10:**
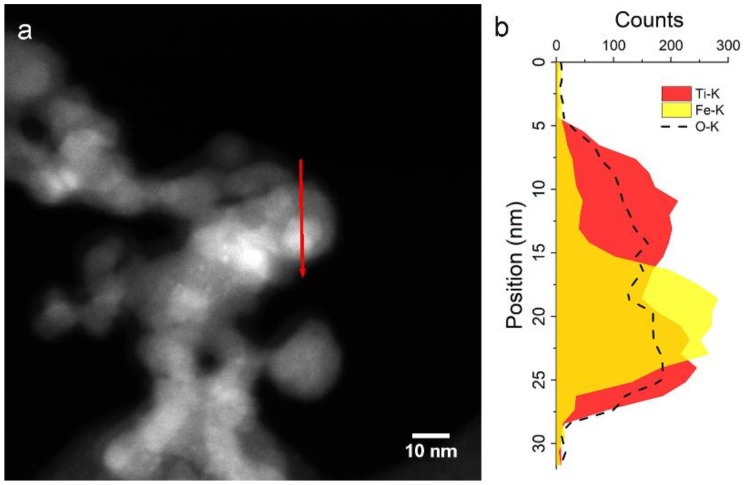
Fe/TiO_x_ sample after 4 h in 1 MPa H_2_ at 400 °C. (**a**) STEM image; (**b**) EDX line profile acquired along the red arrow, highlighting a Fe NP among TiO_x_ NPs.

After a thermal treatment of 4 h at 400 °C under 1 MPa H_2_ (Figure 8b), the intensity of TiO_1-δ_ Bragg reflections decreases significantly, while broad peaks attributable to rutile and anatase TiO_2_ become visible. The crystallite size of α-Fe estimated from the breadth of XRD peaks is almost unaffected by the treatment, passing from 18±2 nm of the as-prepared sample to 21±2 nm. The STEM-EDX scan profile in Figure 10 shows an α-Fe NP of about 13 nm in contact with TiO_x_ NPs.

**Figure 11:**
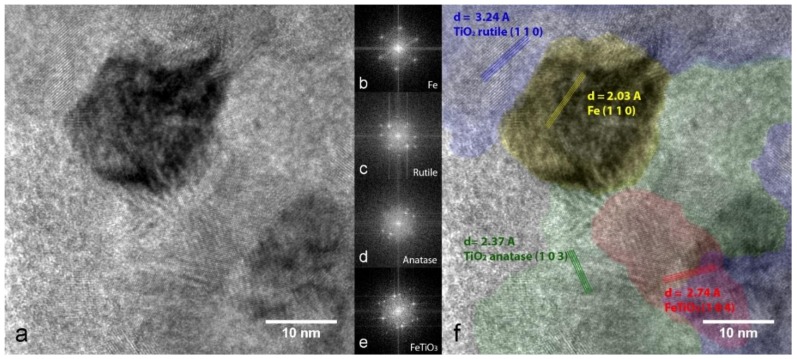
(**a**) HR-TEM image of the Fe/TiO_x_ sample after 24 h in 1 MPa H_2_ at 400 °C; (**b**,**e**) FFTs performed in selected areas as indicated in the crystalline phase distribution map in (**f**), which highlights the lattice planes of α-Fe (yellow) rutile (blue), anatase (green) and ilmenite (red).

When the treatment is prolonged for 24 h, the XRD pattern clearly displays anatase and rutile TiO_2_ Bragg reflections (Figure 8c) along with some residual TiO_1-δ_. In addition, the presence of a small amount of ilmenite FeTiO_3_ is detected. The narrowing of the α-Fe peaks corresponds to an increased average crystallite size of about 30 nm. The HR-TEM image and phase mapping displayed in Figure 11 show an α-Fe NP in contact with both rutile and anatase, together with ilmenite in the interfacial region.

The different oxidation thermodynamics of Fe and Ti explains the results of XRD. We already pointed out that oxidation of TiO_1-δ_ into TiO_2_ is expected during the treatment due to water impurities. Considering now Fe, Equations (1) and (2) must be rewritten as:

(3) $$(3/4)\mathrm{Fe}\;+\;H_{2}O\;↔\;(1/4){\mathrm{Fe}}_{3}O_{4}\;+\;H_{2}$$

(4) $${\left(\frac{P_{H2O}}{P_{H2}}\right)}_{eq}=\exp\left(\frac{\Delta H_{Fe3O4}-\Delta H_{H2O}}{2RT}\right)$$

where $\Delta H_{Fe3O4}=-551$ kJ/mol O_2_ is the enthalpy of the reaction (3/2)Fe + O_2_→(1/2)Fe_3_O_4_. With these data and T = 673 K, equation (4) yields ${\left(P_{H2O}/P_{H2}\right)}_{eq}\approx 7\cdot 10^{-3}$. Since the water content in the H_2_ atmosphere is likely below this value, reaction (3) proceeds leftwards, i.e., any Fe_3_O_4_ at the surface of the NPs should be reduced during the treatment. We notice that also the amount of Fe_3_O_4_ in the as-prepared sample is below the detection limit of XRD. It is possible that the contact with TiO_1-δ_ protects Fe NPs from oxidation during air exposure. In the case of Fe-Co alloy or pure Co NPs, the amount of oxide is expected to be even lower because the enthalpy of Co oxidation is slightly less negative compared to Fe, i.e., $\Delta H_{Co3O4}=-479$ kJ/mol O_2_ and $\Delta H_{CoO}=-491$ kJ/mol O_2_.

**Figure 12:**
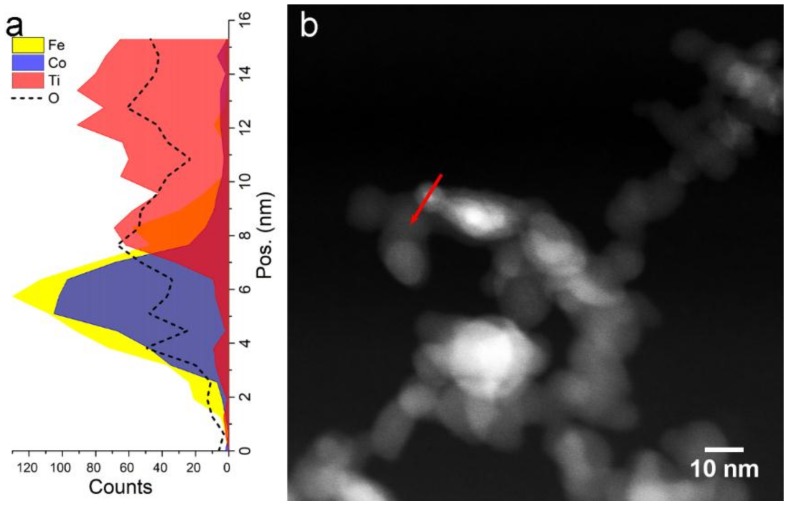
(**a**) EDX profile acquired along the path indicated with a red arrow in the corresponding STEM image (**b**) of the Fe_50_Co_50_/TiO_x_ sample showing a Fe-Co NP in contact with TiO_x_ NPs.

**Figure 13:**
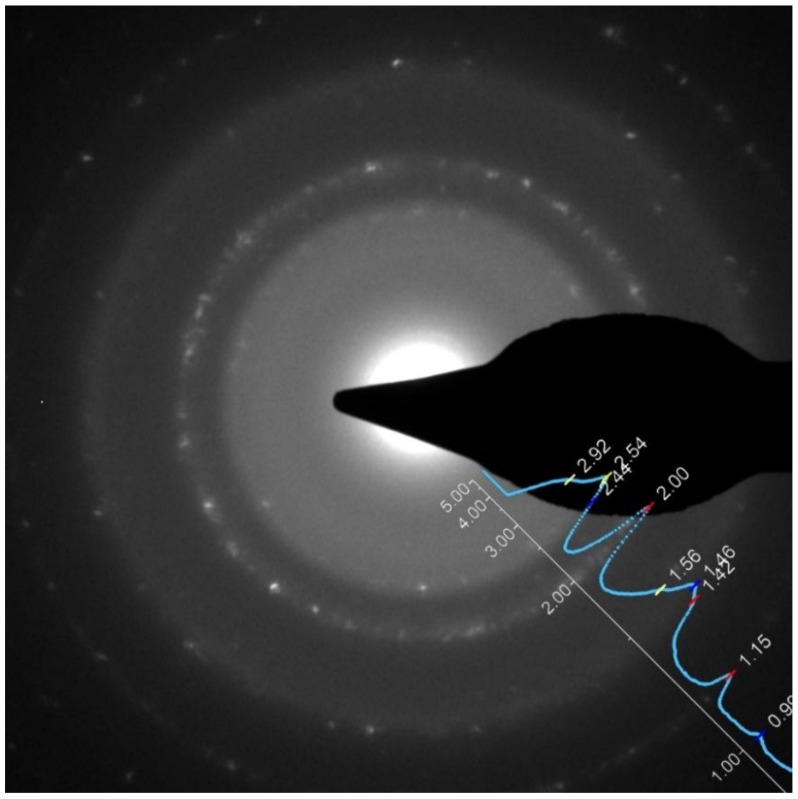
SAD acquired for the Fe_50_Co_50_/TiO_x_ as-prepared sample. The overlaid blue plot is the profile analysis of the SAD obtained by azimuthal integration of the underlying image. The profile was computed by using the plugin PASAD [38] for the software Digital Micrograph from Gatan. The x-axis represents the interplanar spacing in Å. The position of Bragg reflections are indicated by the vertical colored bars and are labeled with the corresponding *d*-spacing. TiO_1-δ_, α-Fe-Co alloy, and cobalt ferrite (blue, red and yellow vertical bars respectively) are detected.

From the point of view of NPs mixing and TiO_x_ stoichiometry, the scenario in Fe_50_Co_50_/TiO_x_ is analogous to Fe/TiO_x_. Figure 12 shows an EDX profile recorded for the as-prepared Fe_50_Co_50_/TiO_x_ sample, in which a small Fe-Co NP with a diameter of about 7 nm appears supported on a Ti-rich NP. According to the previous discussion for Fe-Co NPs and Fe/TiO_x_ NCs, we may expect that Fe_50_Co_50_ NPs crystallize in the BCC α-phase with some cobalt ferrite, while Ti-rich NPs develop mainly in the TiO_1-δ_ phase. The phase analysis by electron diffraction supports this view, as demonstrated in Figure 13. In fact, the SAD azimuthal integration proves that the Fe_50_Co_50_/TiO_x_ sample is composed by α-Fe-Co, cobalt ferrite and TiO_1-δ_. Cobalt ferrite is associated with the broad halos in the SAD pattern. This is compatible with the formation of a 2–3 nm thick oxide shell surrounding the metallic core, as shown in Section 3.2 for Fe-Co NPs.

A certain degree of NPs aggregation is typical of gas-phase condensation processes. If the NPs density in the gas phase is low (i.e., at low evaporation rates), aggregation takes place mainly on the collection surface. Welding of NPs is driven by capillary forces that act to reduce the surface free energy. At high evaporation rates, aggregation takes place also in the gas phase. The typical size of the aggregates is in the hundreds of nanometer range and their shape is generally much ramified. In elements with low melting points such as Mg, aggregation may even result in single crystal NPs [37]. In the presented metal/oxide nanocomposites, the oxide NPs seems to prevent aggregation of the metallic ones, as long as these are kept at a small volume fraction. This should allow to exploit interesting properties of individual NPs such as plasmonic resonance and superparamagnetism.

## 4. Conclusions

We have presented a novel synthesis method for the preparation of metal/oxide nanocomposites based on the physical assembly of nanoparticles (NPs), which are formed by gas phase condensation in a He/O_2_ atmosphere. This approach goes beyond the simple post-synthesis partial oxidation of elemental [33,34] or alloy [39,40] NPs that can only yield a metal core-oxide shell morphology with obvious compositional restrictions. Indeed, our strategy has the potential to provide greater versatility in terms of both composition and independent control over the NPs size and morphology of the two phases.

The synthesis method was demonstrated here for Fe/TiO_x_ and Fe-Co/TiO_x_ nanocomposites, but can be extended to other metal/oxide combinations and may benefit from peculiar features of gas phase condensation, some of which were not explored in the present work. These include: (i) good nanoscale mixing can be achieved also in case of immiscible precursors [41]; (ii) the size of metal NPs can be controlled by tuning the evaporation rate and the inert gas pressure [37]; homogeneous alloy NPs can be synthesized provided that the evaporation rate of the elements are similar, for example Fe-Co, Fe-Ni, Co-Ni, Ag-Au, Au-Cu; NPs sources based on high-pressure sputtering can be employed for the synthesis of refractory metal and oxide NPs [39]; the NPs assembly can be compacted in situ to produce dense pellets with varying degrees of porosity [42]. Obtaining a metal/oxide nanocomposite from the evaporation of two metallic precursors requires that they exhibit strongly different oxidation enthalpies. Besides the case of Ti explored here, we envisage that other suitable precursors for the formation of oxide NPs may be Mg, Al, and Si, all having an oxidation enthalpy more negative than −900 kJ/mol O_2_. These may be combined with NPs of late transition metals, including noble metals. Post-synthesis thermal treatments in a suitable atmosphere permit to control the stoichiometry of the oxide NPs to a certain extent, reducing at the same time the oxidized surface shell around metal NPs. Future work will explore other metal/oxide combinations and characterize their physical/chemical properties.

## Figures and Tables

**Table 1:** Gas flow and O_2_ partial pressure during the synthesis of TiO_x_ NPs. The total pressure was 260 Pa. The samples were examined as-prepared and after being subjected to different thermal treatments as indicated.

Sample	Inlet Flow [nmL/min]		O_2_ Partial P [Pa]	Post-Synthesis Treatment (P = 0.1 MPa, T = 400 °C)		
	He	O_2_		H_2_	Ar	air
Ti-O_l	60.0	0.1	0.4	x	x	x
Ti-O_h	60.0	0.5	2.2			x

**Table 2:** Fe-Co alloy NPs. Comparison between the composition of the precursor powder mixture and of the synthesized NPs. The samples names reflect the NPs composition as determined by SEM-EDX.

Sample	Fe-Co Precursor Mixture [at%]		Fe-Co NPs [at%]	
	Fe	Co	Fe	Co
Fe_100_	100	0	100	0
Fe_77_Co_23_	76(1)	24(1)	77(1)	23(1)
Fe_52_Co_48_	51(1)	49(1)	52(1)	48(1)
Fe_32_Co_68_	27(1)	73(1)	32(1)	68(1)

**Table 3:** Gas flow and O_2_ partial pressure during the synthesis of Fe-Co/TiO_x_ NCs. The total pressure was 260 Pa. The Co and Fe content in the mixed powder precursor are reported.

Sample	Inlet Flow [nmL/min]		O_2_ Partial P [Pa]	Fe-Co Precursor Mixture [at%]	
	He	O_2_		Fe	Co
Fe/TiO_x_	60.0	0.1	0.4	100	-
Fe_50_Co_50_/TiO_x_	60.0	0.1	0.4	50(1)	50(1)

**Table 4:** Quantitative phase analysis for TiO_x_ NPs samples after thermal treatments: rutile vs anatase abundance (wt%), crystallite size *d* and lattice parameters *a* and *c*.

Treatment Atmosphere	Anatase TiO_2_				Rutile TiO_2_			
	wt%	*d* [nm]	*a* [Å]	*c* [Å]	wt%	*d* [nm]	*a* [Å]	*c* [Å]
Ti-O_l								
H_2_	56(1)	16(2)	3.7895(3)	9.465(2)	44(1)	11(1)	4.5970(7)	2.9568(8)
Ar	64(1)	14(2)	3.790(1)	9.459(2)	36(1)	8(1)	4.600(1)	2.956(2)
air	74(2)	15(2)	3.7855(3)	9.461(2)	26(2)	8(2)	4.597(2)	2.945(2)
Ti-O_h								
air	84(1)	13(1)	3.798(2)	9.4691(4)	16(1)	6.0(5)	4.595(6)	2.945(6)

**Table 5:** Crystallite size *d* and lattice parameter *a* of the three crystalline phases identified by XRD in Fe-Co NPs. The FCC γ-phase is only observed at the highest Co content.

Sample	α-Fe-Co		Cobalt Ferrite		γ-Fe-Co	
	*d* [nm]	*a* [Å]	*d* [nm]	*a* [Å]	*d* [nm]	*a* [Å]
Fe	15(1)	2.8729(4)	2.0(2)	8.451(6)	-	-
Fe_77_Co_23_	19(2)	2.8686(2)	2.7(2)	8.4272(6)	-	-
Fe_52_Co_48_	18(2)	2.8629(1)	2.4(2)	8.442(4)	-	-
Fe_32_Co_68_	24(2)	2.8406(2)	n.d.	n.d.	10(2)	3.564(2)
